# Supplementary material for: Green Synthesis of Gold, Silver, Copper, and Magnetite Particles Using Poly(tartaric acid) Simultaneously as Coating and Reductant
Source: Polymers (Basel). 2023 Nov 21;15(23):4472. doi: 10.3390/polym15234472 (PMC10708409; doi:10.3390/polym15234472)
Supplement: Supplementary file 1 [file polymers-15-04472-s001.zip › polymers-2727253-supplementary.pdf]

polymers

MDPI

Supporting Information for:

Green Synthesis of Gold, Silver, Copper, and Magnetite Particles Using Poly(Tartaric Acid) Simultaneously as Coating and Reductant

Alexander Bunge [1], Teodora Radu [1], Gheorghe Borodi [1], Sanda Boca [1,2,†] and Alexandrina Nan [1,*]

Synthesis of sodium polytartrate **7***:*

Poly(tartaric acid) **1** (2.00 g) was heated in water (15 mL), and sodium hydrogencarbonate (1.68 g) was added. After the polymer was dissolved, ethanol (100 mL) was added, and the supernatant was decanted. After adding more ethanol (100 mL), sonicating and decanting, the remaining solvent was evaporated and the solid dried in at 60 °C over night to obtain 2.34 g **7** as a tan powder.

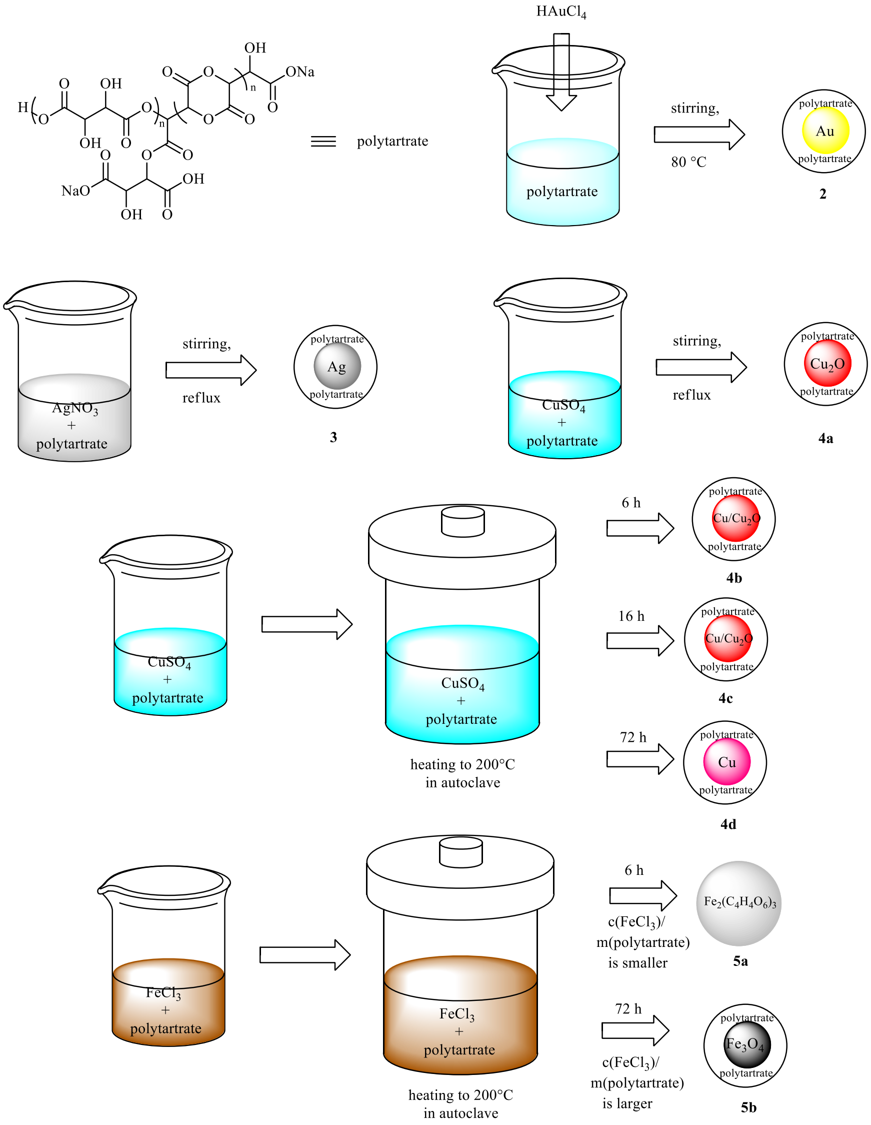

Scheme S1. Schematic view of the syntheses of gold, silver, copper/copper oxide, ferrous tartrate and magnetite particles

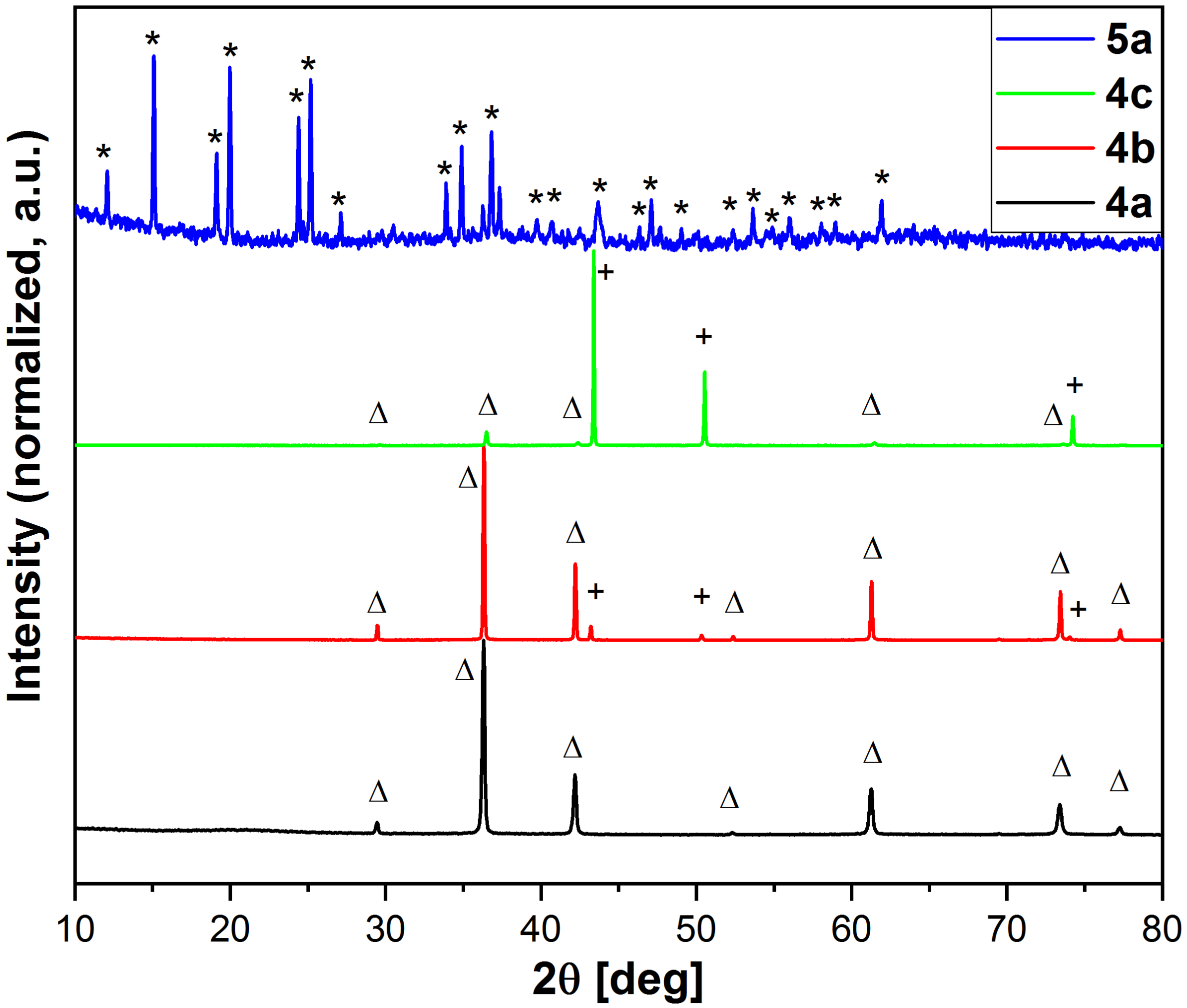

Figure S1. XRPD of samples **4a-c** and **5a**. The reflection peaks are labelled *: ferrous tartrate (PDF 01-0347); +: copper (PDF 89-2838); Δ: cuprite (PDF 78-2076)

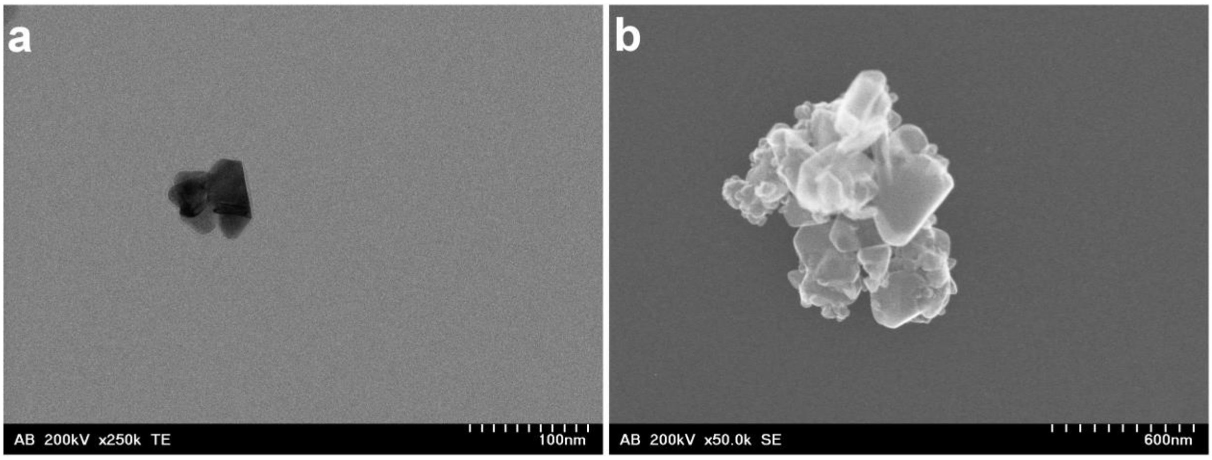

Figure S2. Additional (**a**) TEM and (**b**) SEM of sample **3**

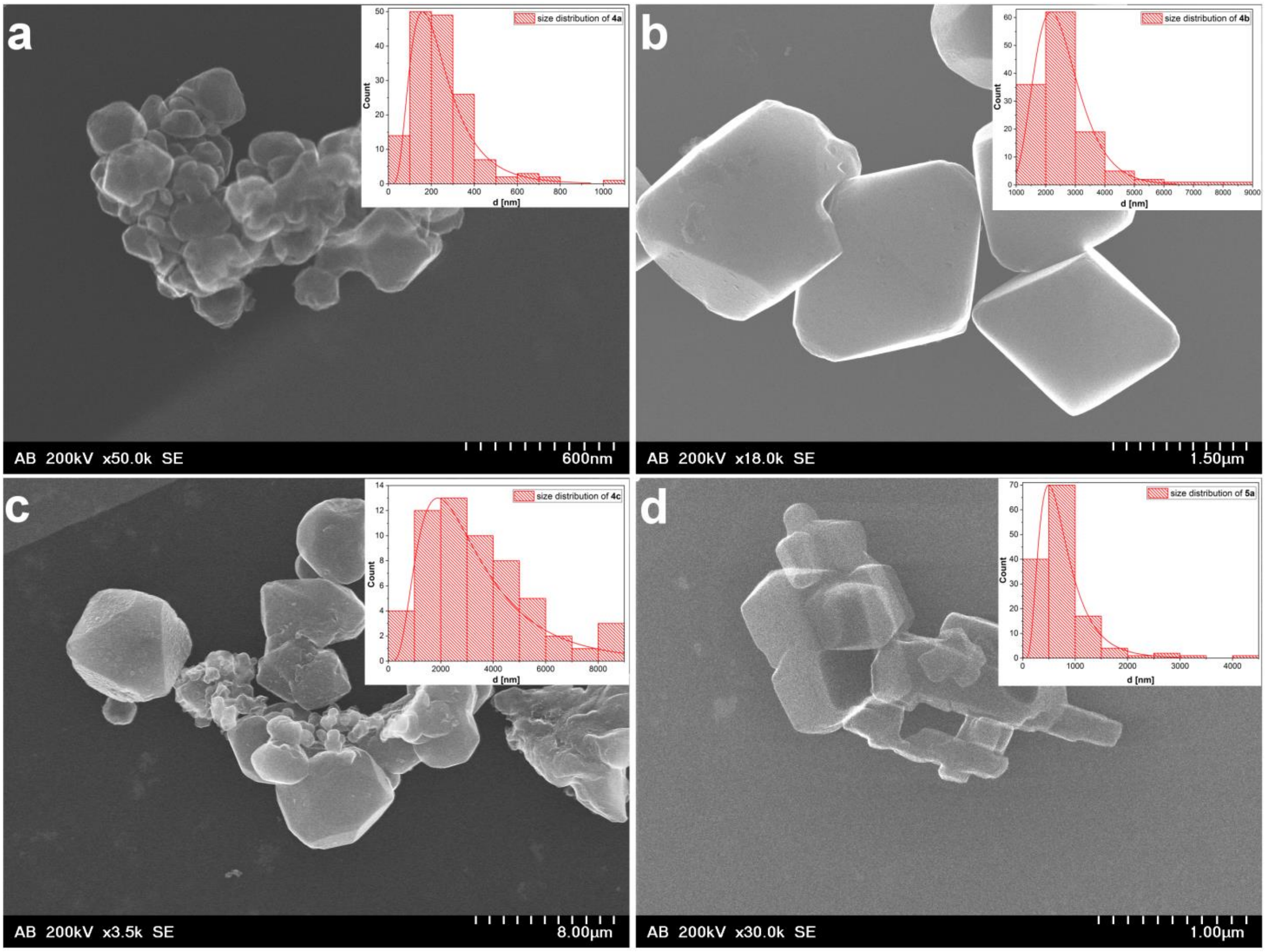

Figure S3. SEM of (**a**) **4a**, (**b**) **4b**, (**c**) **4c** and (**d**) **5a**. Inset: Size distributions of the particles.

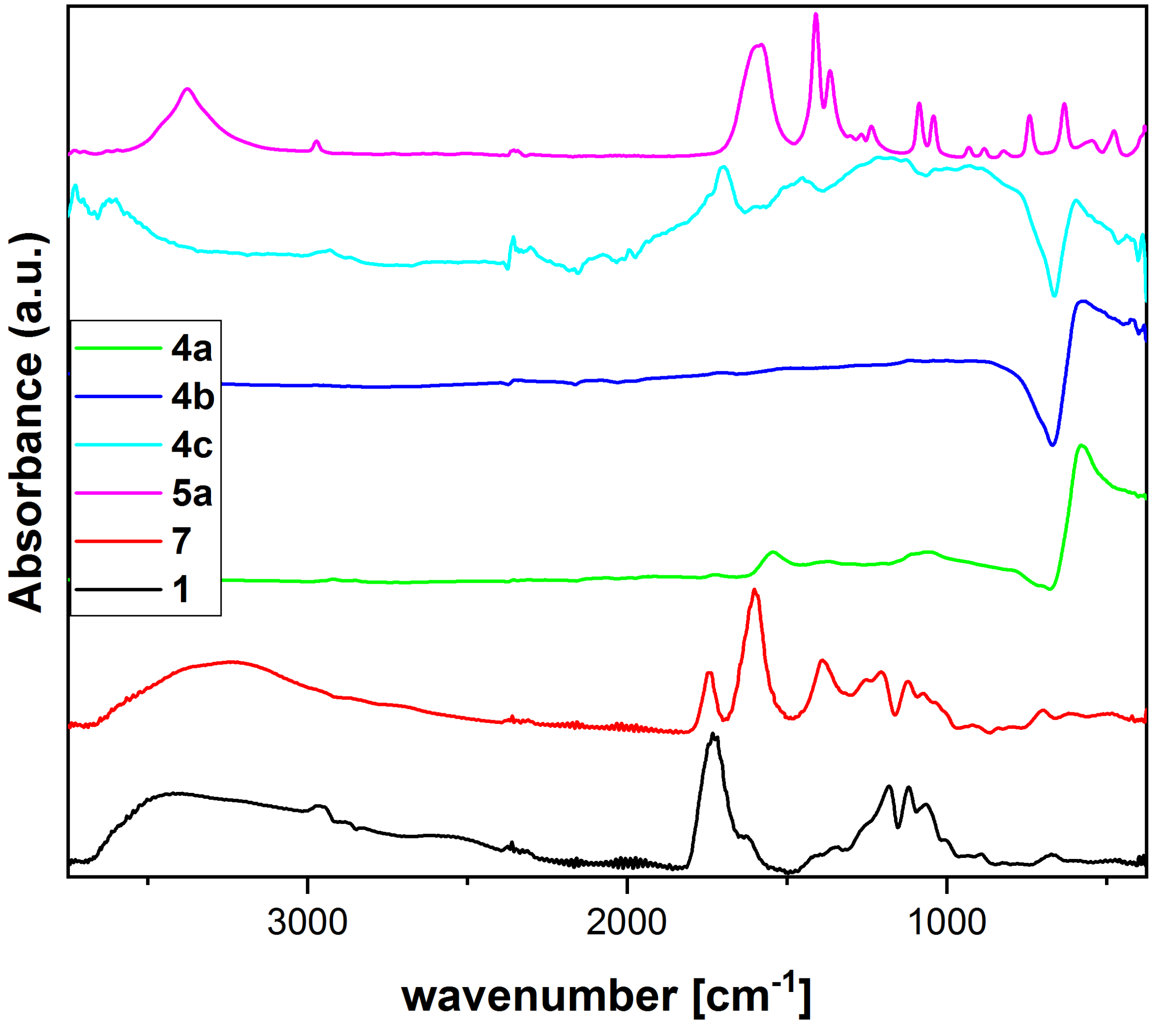

Figure S4. FTIR spectra of **1**, **4a**, **4b**, **4c**, **5a** and **7.**

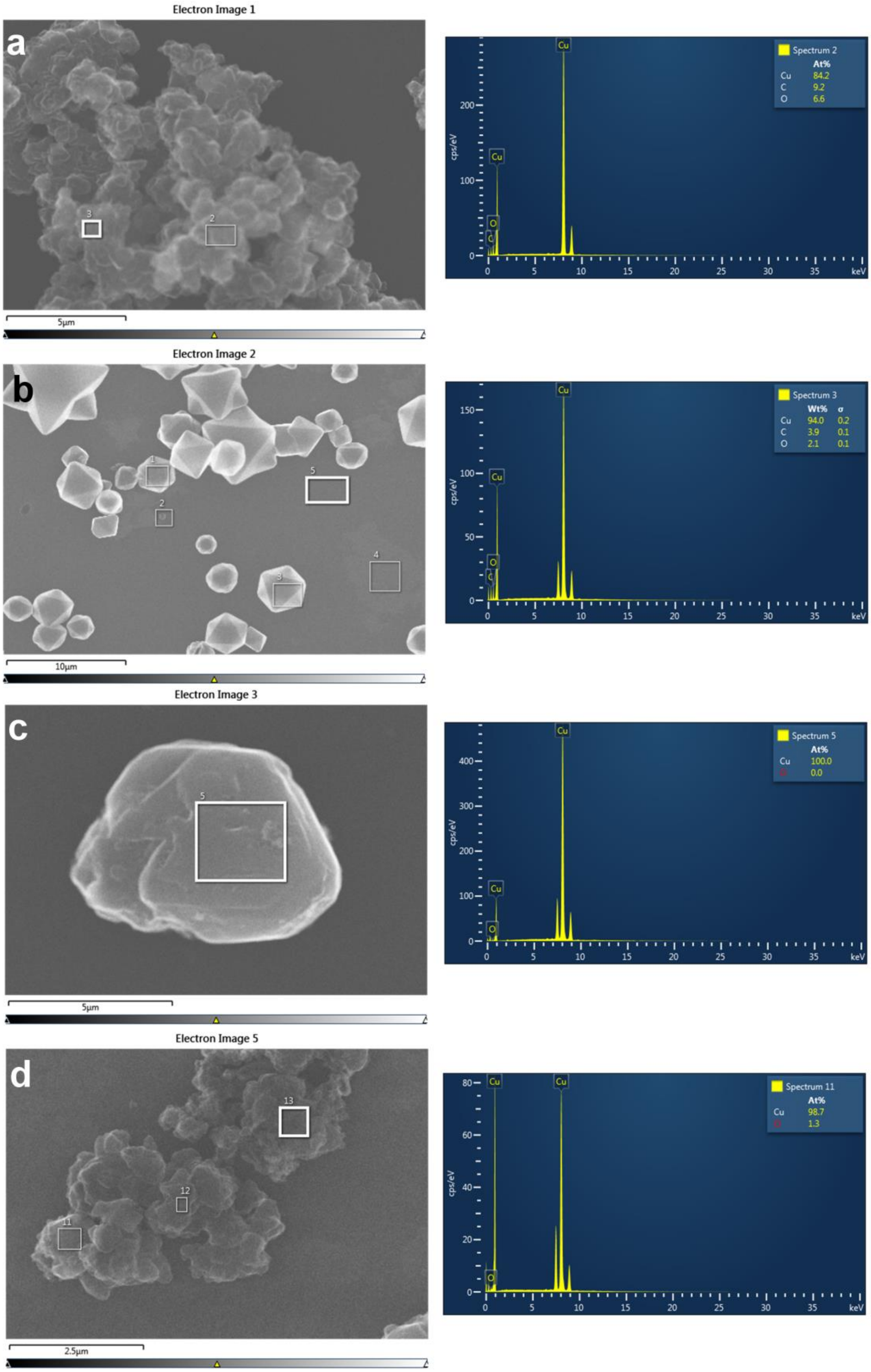

Figure S5. EDX of (**a**) **4a**, (**b**) **4b**, (**c**) **4c** and (**d**) **4d.**

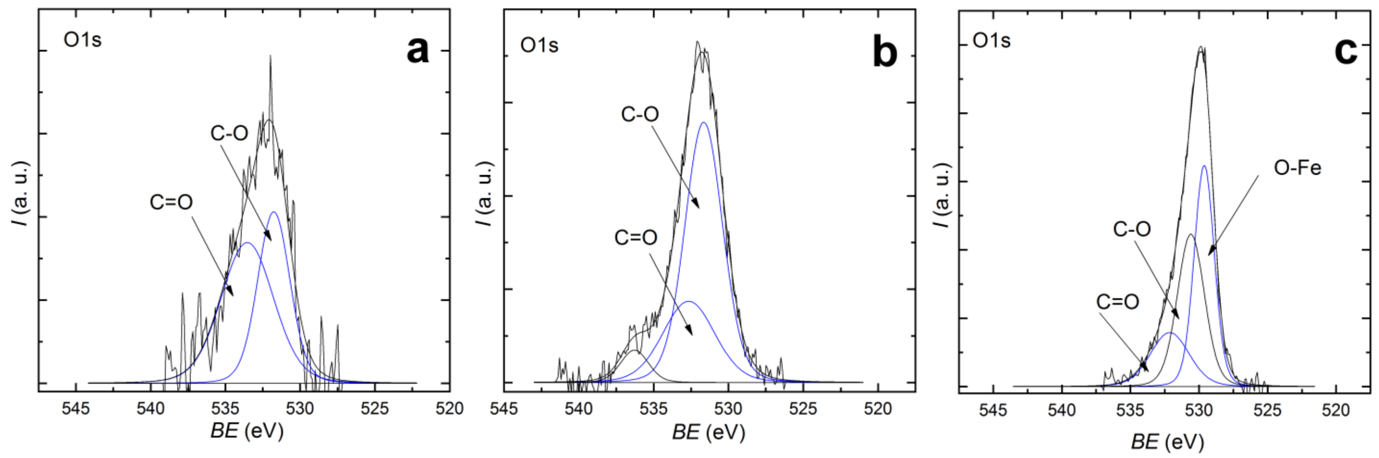

Figure S6. XPS O1s core level spectra of **3** (**a**), **4d** (**b**) and **5b** (**c**).

Table S1. Size distributions determined by TEM.

Sample	Size (Determined by TEM) [nm]
**2**	35 ± 11
**3**	42 ± 30
**4a**	270 ± 168
**4b**	2580 ± 1050
**4c**	3390 ± 2060
**4d**	723 ± 372
**5a**	793 ± 555
**5b**	18 ± 4

Table S2. Thermal conductivity, diffusivity and volumetric heat capacity of samples **1**, **6a**, **6b** and **6c** at room temperature and 50 °C.

Sample (Temperature)	Thermal Conductivity [W/mK]	Diffusivity [mm^2/s]	Volumetric Heat Capacity [MJ/m^3K]
**1** (RT)	0.245	0.219	1.117
**6a** (RT)	0.218	0.1	2.173
**6b** (RT)	0.211	0.2	1.051
**6c** (RT)	0.229	0.262	0.876
**1** (50 °C)	0.249	0.227	1.104
**6a** (50 °C)	0.248	0.356	0.833
**6b** (50 °C)	0.204	0.259	0.789
**6c** (50 °C)	0.224	0.349	0.641
